# ASOG: AntiSense Oligonucleotide Generator

**DOI:** 10.1016/j.csbj.2025.09.022

**Published:** 2025-09-16

**Authors:** Jonah Kimi, Patricia Korczak, Brune Vialet, Eric Roubin, Philippe Barthélémy, Sébastien Campagne, Florian Malard

**Affiliations:** a Univ. Bordeaux, CNRS, INSERM, ARNA UMR 5320, U1212, F-33000 Bordeaux, France; b Univ. Bordeaux, CNRS, INSERM, IECB, US1, UAR 3033, F-33600 Pessac, France

**Keywords:** Antisense oligonucleotide, Melting temperature calculation, Splice site prediction, BLASTn, Webserver

## Abstract

Antisense oligonucleotides (ASOs) are used in both fundamental research and clinical applications to modulate gene expression by targeting the RNA transcript of specific genes. Historically, ASOs were designed manually, a time-consuming process that limited exhaustive searches through the ASO space. More recently, resources have been developed based on traditional or deep learning approaches to facilitate ASO design, each with their specific use cases and limitations. In this context, we propose an original and generalistic pipeline for ASO design, based on explicit criteria, original algorithms, and third-party software, encapsulated in a web application we named AntiSense Oligonucleotide Generator (ASOG). The ASOG pipeline requires only a target gene sequence as input, and it proceeds with ASO generation, predicts the structural properties of target subsequences, predicts splice site masking, detects off-target effects, and computes thermodynamic hybridization parameters, taking into account some of the most common RNA modifications. ASOG is designed to enable users to quickly navigate the ASO space, assisting them in making informed decisions. The ASOG webserver is available at asog.iecb.u-bordeaux.fr.

## 1 Introduction

Antisense oligonucleotides (ASOs) are short nucleic acid sequences used to modulate gene expression in both fundamental research and therapeutic applications [1]. ASOs are designed to target specific regions within pre-mRNA or mature mRNA, thereby triggering transcript degradation [2], altering splicing patterns [3], or modulating translation [4], among other mechanisms. Their clinical relevance is particularly significant in the context of monogenic disorders [5]. Notable examples include nusinersen (Spinraza ®) [6] and eteplirsen (Exondys 51 ®) [7], which are used for the treatment of Spinal Muscular Atrophy (SMA) and Duchenne Muscular Dystrophy (DMD), respectively. Moreover, the biological activity of ASOs depends, in part, on sequence complementarity with the target. This relatively straightforward pharmacological principle enables the targeting of genes that were previously considered undruggable [8]. However, designing biologically effective ASOs remains a complex task, as it requires consideration of multiple factors, including but not limited to target stability, hybridization thermodynamics, off-target effects, and chemical modifications to improve the pharmacological properties of the drug candidate.

Historically, ASOs were designed manually through a process that involved establishing a set of candidate sequences and systematically assessing their properties using third-party tools, prior to candidate prioritization and experimental evaluation. Due to the time-consuming nature of this task, this approach does not allow for exhaustive exploration of the ASO space for a given target gene sequence. To facilitate the design of biologically active ASOs, several computational tools have been developed, some of which are freely available online. These include ASOptimizer [9] to optimize the chemical diversity of antisense oligonucleotides, PFRED [10] for the development of RNase H-mediated ASOs, the MASON [11] webserver for bacterial ASO discovery, eSkip-Finder [12] for exon-skipping ASOs, AOBase for database-oriented ASO design and selection [13], among others. These tools may rely on machine learning models, implicit selection criteria, or database searches to inform users. Although they demonstrate effectiveness for their intended purposes, these tools are not designed to perform an exhaustive search of the ASO space for a given target gene sequence, nor to provide a comprehensive evaluation of oligonucleotide properties, including target secondary structure, splice site prediction, hybridization thermodynamics, self-folding potential, and off-target binding.

To complement the existing suite of tools for ASO design, we developed the Antisense Oligonucleotide Generator (ASOG), a general-purpose web application for automated ASO definition and property assessment. ASOG integrates a modular pipeline that combine well-established computational tools with custom algorithms to generate and annotate ASO sequences for any user-provided target gene. By enabling an exhaustive search of ASO sequences, the web server computes explicit criteria relevant to ASO selection, including target RNA topology, melting temperature, splice site masking potential, self-folding properties, and off-target interactions. ASOG also offers standalone modules for melting temperature prediction and splice site analysis, providing the research community with broadly useful tools beyond ASO design.

Here, we describe the development, implementation, and validation of ASOG. We illustrate its functionality using a test case on β-thalassemia, demonstrating that the platform can reproduce known active ASOs targeting the *HBB* IVS2^654^ mutation [14], [15]. By providing an accessible, transparent, and modular platform, ASOG facilitates systematic exploration of ASO space and supports informed decision-making in both basic research and therapeutic discovery. Our web application ASOG is freely available online at asog.iecb.u-bordeaux.fr.

## 2 Methods

### 2.1 Development and environment

ASOG was developed on GNU/Linux Debian 11 (Bullseye) and is deployed in production on Ubuntu 22.04.5 LTS. The back end of the application is built with Python (v3.12) using the high-level Django web framework (v4.2.15), while user data are stored in MongoDB (v8.0.1). The ASOG production server relies on Nginx (v1.18.0), which functions as both a content delivery network and a reverse proxy, implementing Transport Layer Security (TLS 1.2) and Server Name Indication (SNI) protocols for HTTPS-only compliance. Nginx forwards HTTPS requests as HTTP to Gunicorn, which uses WSGI to interface with Django. The front-end interface is developed with HTML5 and CSS3 (using Bootstrap v4.1.3), together with JavaScript libraries such as jQuery, CanvasJS, Forna [16], and BlasterJS [17] to implement dynamic functionality.

### 2.2 Third-party software

ASOG integrates third-party software components both within the ASO generation pipeline and as standalone tools accessible through the web server. To predict nucleic acid topology, ASOG relies on a locally installed version of the well-established Mfold tool [18], which has been slightly modified to accept input sequences of up to 9000 nucleotides. To predict the localization of donor (5’) and acceptor (3’) splice sites in target gene sequences, we use a local installation of SpliceAI [19], a 32-layer deep convolutional neural network model recognized for its high accuracy in splice site prediction based on sequence context. For each generated ASO, specificity and off-target potential are assessed using a local installation of the NCBI BLASTn software [20], with searches performed against nucleotide databases from several species, including *Homo sapiens* and *Mus musculus*.

### 2.3 Melting temperature calculation

ASOG features a *TmCalc* module that calculates the melting temperature of nucleic acid duplexes. This module is based on the methods from the established OligoCalc tool [21]. We provide the specific equations employed by ASOG for each method and scenario below.

#### 2.3.1 Nearest neighbor (NN) model

The NN model, preferred for calculating melting temperatures, relies on thermodynamic principles [21], [22], [23]. It uses thermodynamic data from experiments on DNA/DNA [23], RNA/RNA [24], RNA/Phosphorothioate RNA [24], and RNA/2’-O-methylated RNA [25] duplexes.

For duplexes with strands of 8 nucleotides or longer:

(1) $$T_{m}=\frac{\sum (\Delta H_{d})-3.4}{\sum (\Delta S_{d})+R\log_{2}\left(\frac{1}{\left[duplex\right]}\right)}+272.9+16.6\log_{10}[Na^{+}]$$

In Eq. (1), $\Delta H_{d}$ and $\Delta S_{d}$ represent the sums of enthalpy and entropy across all nearest-neighbor doublets, respectively. The term $16.6\log_{10}([Na^{+}])$ modifies the $T_{m}$ based on salt concentration variations. This equation assumes standard annealing conditions at pH 7.0.

#### 2.3.2 Basic method

The basic melting temperature calculation is the historical method but is the least preferred, provided as a baseline for comparison [21], [26], [27].

For sequences of 13 nucleotides or fewer:

(2) $$T_{m}=(wA+xT)\times 2+(yG+zC)\times 4$$

For sequences of 14 nucleotides or longer:

(3) $$T_{m}=64.9+41.0\left(\frac{yG+zC-16.4}{wA+xT+yG+zC}\right)$$

In (2), (3), the variables w, x, y and z represent the number of bases A, T, G, and C, respectively. Both equations assume that annealing occurs under the standard conditions of 50 nM primer, 50 mM Na^+^, and pH 7.0.

#### 2.3.3 Salt-adjusted basic method

The salt-adjusted basic method accounts for changes in salt concentration in melting temperature calculation [21], [28].

For DNA sequences of 13 nucleotides or fewer:

(4) $$\begin{array}{ll} T_{m} & \;=(wA+xT)\times 2+(yG+zC)\times 4-16.6\log_{10}(0.05)\\ & \;+16.6\log_{10}([Na^{+}]) \end{array}$$

For DNA sequences of 14 nucleotides or longer:

(5) $$\begin{array}{ll} T_{m} & \;=100.5+41\left(\frac{yG+zC}{wA+xT+yG+zC}\right)-\left(\frac{820}{wA+xT+yG+zC}\right)\\ & \;+16.6\log_{10}([Na^{+}]) \end{array}$$

For RNA sequences:

(6) $$\begin{array}{ll} T_{m}= & \;79.8+18.5\log_{10}([Na^{+}])+58.4\left(\frac{yG+zC}{wA+xT+yG+zC}\right)\\ & \;+11.8{\left(\frac{yG+zC}{wA+xT+yG+zC}\right)}^{2}-\frac{820}{wA+xT+yG+zC} \end{array}$$

In Eq. (4) the term $log_{10}(0.05)$ adjusts for the salt concentration of 50 mM Na^+^. In (4), (5), (6), the variables w, x, y and z represent the number of bases A, T, G, and C, respectively, and the term $16.6log_{10}([Na^{+}])$ adjusts the $T_{m}$ for changes in the salt concentration. These equations assume annealing under standard conditions of 50 nM primer and pH 7.0.

## 3 Results

### 3.1 Overview

The ASOG web application is designed to provide straightforward workflows through a clean and user-friendly interface. Anonymous users may access standalone tools that operate independently of the main ASO generation pipeline. Access to the pipeline itself requires user registration, which provides each user with a dedicated profile and job history while also reducing the risk of server misuse through anonymous requests. All data generated by ASOG can be visualized online and downloaded in multiple formats, including tables and figures. For every tool, each user-defined parameter is documented on a dedicated page, and explicit credit is given to third-party software integrated into the application. ASOG is, and will remain, free of charge for academic and non-commercial use.

### 3.2 Standalone tools

The ASO generation pipeline implemented in ASOG includes broadly useful modules, which we have made available as standalone tools for splice site prediction and melting temperature calculation.

#### 3.2.1 Splice site prediction

Splice site prediction is of broad interest to the RNA splicing community, particularly for altering splicing patterns for both fundamental research and therapeutic purposes. We have integrated SpliceAI [19], a deep learning model that predicts splicing from pre-mRNA sequences, into ASOG. The tool is freely accessible without registration at asog.iecb.u-bordeaux.fr/spliceai. This convolutional neural network accurately predicts both canonical and cryptic splice sites, as well as mutations that affect splicing, which are relevant to a wide range of disorders and can inform the design of splice site-masking oligonucleotides [19], [29], [30]. Users provide a nucleotide sequence as input, and the model analyzes it within a broad sequence context to capture long-range interactions and predict splicing outcomes with high accuracy.

#### 3.2.2 Melting temperature calculation

Melting temperature calculation is a common task in molecular biology. Online tools such as OligoCalc provide estimates for DNA/DNA and RNA/RNA homoduplexes [21], but not for heteroduplexes involving chemically modified RNAs. To address this limitation, we developed the *TmCalc* module within the ASOG web application, accessible without registration at asog.iecb.u-bordeaux.fr/tmcalc. This tool calculates melting temperatures for both homoduplexes and heteroduplexes, including those formed between unmodified RNA and chemically modified RNAs such as phosphorothioate (PS) or 2’-O-methylated (2’OMe) RNA. Calculations are based on established methods and published thermodynamic parameters, employing the Nearest-Neighbor (NN) model and specific datasets for diverse nucleic acid duplexes [22], [23], [24], [25].

### 3.3 Pipeline for ASO generation

**Fig. 1:**
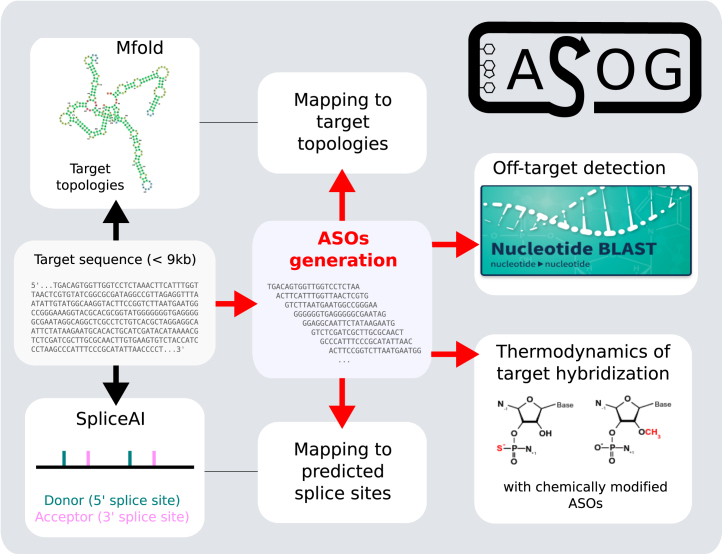
**Automated oligonucleotide design and property assessment with ASOG.** The ASOG pipeline generates ASOs and evaluates their properties as follows: First, it assesses the topology of the target sequence using Mfold [18] and splice sites with SpliceAI [19]. It then generates overlapping ASOs mapped to target subsequences. Off-target interactions are checked using the BLASTn program [20]. Finally, it computes self-folding properties and hybridization thermodynamics, including the melting temperature of heteroduplexes with chemically modified RNAs.

The core functionality of the ASOG web application is its ASO generation tool, which is available to registered users at asog.iecb.u-bordeaux.fr/generate_asos (Fig. 1). This tool requires users to input a target gene sequence, which is then processed to generate a dataset of ASOs with properties relevant to the user. The ASO generation pipeline was designed to enable a rapid and exhaustive search through the ASO space, helping users save time and make more informed decisions in their ASO design tasks.

#### 3.3.1 User input

**Fig. 2:**
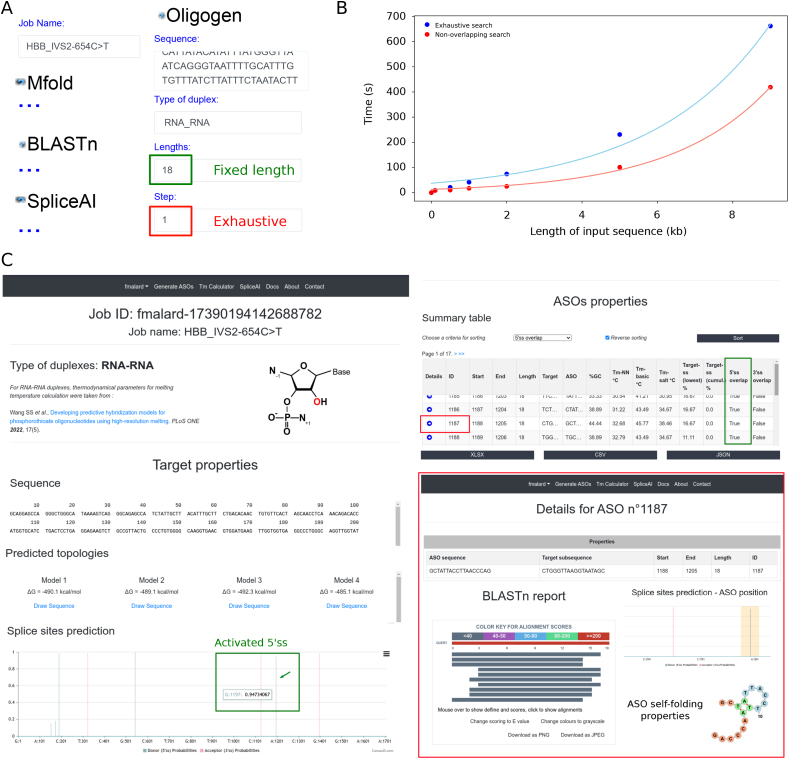
**Overview of the *Generate ASOs* module.** (A) Abstract of input fields for the *Generate ASOs* module. The job HBB_IVS2^654^C > T aims to identify ASO sequences that correct *HBB* pre-mRNA splicing errors due to a second intron mutation, using a sliding window with a *Step* of 1 and a *Lengths* 18 nucleotides for exhaustive search with fixed length. (B) Execution time for the *Generate ASOs* module. Input sequences of increasing lengths were submitted for either an exhaustive (*i.e.*, *Step*=*1*) or non-overlapping (*i.e.*, *Step*=*Length*) search. A monoexponential fit was applied to the data series for visualization. (C) Result page overview for job HBB_IVS2^654^C > T. Displays job details, gene-related properties, and a paginated table of ASOs, each with an extended details page featuring an interactive BLASTn report, and analyses of self-folding, splice site masking, and hybridization thermodynamics.

On the *Generate ASOs* input page, users can configure parameters for each module of the ASO generation tool, namely *Oligogen*, *TmCalc*, *Mfold*, *BLASTn*, and *SpliceAI* (Fig. 2). New users can simply enter a job name and use the pre-filled form to run a test job. Alternatively, they can enter their own target gene sequence in the designated text area and submit the job. Key parameters include the *Type of duplex* variable, which drives secondary structure prediction and melting temperature calculations, and can take the following values: DNA/DNA, RNA/RNA, RNA/PS-RNA, and RNA/2’OMe-RNA. Users will also frequently adjust the *Lengths* parameter, which specifies a comma-separated list of lengths for ASO generation, and the *Step* parameter, which controls the exhaustiveness of the generated ASO dataset. The *Database* parameter is also commonly modified to select species-specific nucleotide databases for detecting off-target effects in relevant genomes. In addition to these, a variety of other parameters can be adjusted by users, each of which is described in the documentation available at asog.iecb.u-bordeaux.fr/documentation.

#### 3.3.2 Processing of the target gene sequence

The ASO generation pipeline applies a series of operations to the target gene sequence. First, the *SpliceAI* module predicts the locations of donor (5’) and acceptor (3’) splice sites on the target sequence [19]. This information is used to label oligonucleotides that target regions containing predicted splice sites. Second, the *Mfold* module predicts an ensemble of secondary structure topologies for the target sequence [18]. These topologies are used to identify single-stranded regions, either within the lowest-energy model or across aligned topologies. In the latter case, superimposing predicted topologies enables the identification of regions consistently predicted to remain unstructured, and therefore always single-stranded. This information is used to label oligonucleotides according to the fraction of single-stranded nucleotides at their binding site on the target sequence, considering both the lowest-energy topology and an adjustable ensemble of predicted topologies.

#### 3.3.3 Generation of ASOs and properties

The target gene sequence is used to generate ASOs through a sliding-window procedure, with window width and stride defined by the user-specified *Lengths* and *Step* parameters, respectively. This allows for either subsampling or exhaustive exploration of the ASO space (Fig. 2 A). The *SpliceAI* [19] and *Mfold* [18] modules label each oligonucleotide with predicted splice sites and the fraction of single-stranded nucleotides at the binding site. These annotations are particularly useful for designing splice site-masking oligonucleotides and for identifying ASOs targeting regions embedded within secondary structures. The *Mfold* [18] module also evaluates ASO self-folding properties to prevent the selection of sequences prone to forming stable secondary structures that could impair biological activity. Then, the *TmCalc* module calculates the melting temperature of each oligonucleotide relative to its target sequence, accounting for several duplex types, including chemically modified oligonucleotides. Finally, each ASO is screened for off-target effects using the *BLASTn* [20] module against relevant nucleotide databases to ensure specificity, a critical requirement for both basic research and therapeutic ASO applications.

#### 3.3.4 Dataset visualization

Upon job submission, users are first directed to a waiting page, which automatically redirects to a results page upon job completion. The completion time is generally reasonable but increases with the length of the input sequence (Fig. 2 B). Completed jobs can also be retrieved through the user’s profile and job history. The ASO generation pipeline outputs are organized into a summary view for the entire dataset and detailed views for each individual oligonucleotide (Fig. 2 C). The summary view includes the job identifier, job name, duplex type, and an overview of target properties such as the formatted nucleotide sequence, predicted topologies, and splice site predictions. Predicted topologies can be dynamically visualized in a separate tab using the Forna JavaScript library [16], while splice site predictions along the target gene sequence are shown on the main page through an interactive CanvasJS chart. The summary also displays the ASO dataset, presented in a paginated table to maintain interface responsiveness. This table summarizes key properties such as oligonucleotide identifier, sequence, GC content, coordinates, melting temperature, and splice site masking status. Each ASO entry links to a dedicated view that provides detailed information, including self-folding properties, topology visualization, and a per-base accessibility diagram for the target sequence. Users can sort the summary table by multiple criteria and download the dataset in various formats for both manual and automated analyses. In addition, *BLASTn* results are displayed using the interactive BlasterJS tool [17], [20]. For any given oligonucleotide, users can access alignments by selecting its sequence identifier and download the results as either text tables or images.

## 4 Test case

β-thalassemia is a blood disorder caused by reduced or absent production of the beta chains of hemoglobin. The *HBB* gene, which encodes β-globin, contains three exons and two introns. A mutation at nucleotide 654 of the second intron (IVS2^654^ C > T) leads to aberrant splicing of the *HBB* pre-mRNA and has been identified as a cause of β-thalassemia [14]. The IVS2^654^ mutation induces aberrant splicing by activating cryptic 3’ and 5’ splice sites within the same intron. Previous studies have demonstrated that masking these splice sites with ASOs can restore β-globin expression. In this example, we used the *Generate ASOs* module of ASOG to show that the pipeline can reproduce previously published sequences of active ASOs [15].

We retrieved the genomic DNA sequence corresponding to the *HBB* pre-mRNA transcript (GRCh38:11:5225414:5227121:-1) from the Ensembl database [31]. Using the *SpliceAI* tool within ASOG, we accurately predicted the canonical splice sites of the *HBB* transcript [15], [19]. We then introduced the IVS2^654^ C > T mutation into this sequence and submitted it to the *Generate ASOs* module, which performed an exhaustive search for ASOs of fixed length. The run completed in 84 s, consistent with the expected time for a sequence of approximately 2 kb (Fig. 2 A, B). The results page enabled visualization of target topologies and properties, including the activation of a cryptic 5’ splice site at position G1197 following the mutation [14], [15]. Applying the 5’ splice site filtering option in the ASO summary table revealed a series of overlapping ASOs masking the activated 5’ splice site, including ASO n${\;}^{\circ }$1187, which was previously shown to correct aberrant *HBB* splicing caused by the IVS2^654^ C > T mutation [15]. Detailed information for ASO n${\;}^{\circ }$1187, including a BLASTn report and hybridization thermodynamics, was available for download in multiple formats via the user interface. This example illustrates how the *Generate ASOs* pipeline implemented in ASOG can reproduce published results and accelerate the discovery of active oligonucleotides.

## 5 Discussion

In this work, we introduce ASOG, a platform designed to facilitate the design of ASO sequences for both fundamental and therapeutic applications. The purpose of ASOG is to provide the community with a general and transparent tool for ASO design, computing explicit criteria for ASO selection within a modular pipeline that can be applied to any target sequence within a given gene. Among the freely available online tools, most are tailored to specific applications, such as chemical optimization (e.g., ASOptimizer [9]), RNase H–mediated ASOs (e.g., PFRED [10]), bacterial targets (e.g., MASON [11]), or exon skipping (e.g., eSkip-Finder [12]), among others. Although these tools have demonstrated effectiveness, they are limited to their specific scope of use and are not designed to evolve toward more general approaches.

In contrast, the general-purpose systematic exploration of ASO space from any sequence provided by the ASOG pipeline relies on the modular integration of custom algorithms and well-established tools such as SpliceAI [19], Mfold [18], and BLASTn [20]. The thermodynamic module embedded within ASOG also addresses the gap in melting temperature calculations for nucleic acid heteroduplexes composed of modified RNA backbones, such as phosphorothioate and 2’-O-methyl RNAs. Since we are aware that users might be interested in the modules themselves and not only in the ASO generation pipeline, we have made standalone modules available to the broader community. The user experience has also been optimized through the use of modern technologies for web application development, yielding a clean web interface, user accounts, job history, data visualization, and downloads. All data generated by ASOG are provided to enable informed decision-making based on explicit criteria, and in a validation test case on β-thalassemia, the pipeline correctly identified known active ASOs targeting the *HBB* IVS2^654^ mutation [15], demonstrating both reliability and practical utility while accelerating candidate prioritization and reducing trial-and-error.

Although ASOG shows great promise in reducing the time required to design and select ASO sequences with the desired biological activity, the current pipeline has limitations that highlight opportunities for improvement throughout the software’s life cycle. For example, the availability of ASO targets is currently described using base-pairing information computed with Mfold [18]. This approach does not account for potential binding sites for RNA-binding proteins (RBPs) on the target sequence, which can hinder successful ASO binding in a manner similar to target topology. Another important parameter for ASO selection is the melting temperature of the ASO/target duplex. Although we partially addressed this by implementing thermodynamic tables for RNA/PS-RNA and RNA/2’OMe-RNA heteroduplexes, the diversity of nucleic acid modifications relevant to ASO design is much broader. There remains a need for further studies to derive thermodynamic parameters for relevant heteroduplexes involving modified backbones, such as phosphorodiamidate morpholino oligomers (PMOs) or peptide nucleic acids (PNAs), and sugar modifications, such as 2’-O-methoxyethyl (2’MOE) or Locked Nucleic Acids (LNAs), among others.

In terms of future perspectives, the modular architecture of the ASOG pipelines encourages the integration of novel elements aimed at refining computed data for each ASO sequence, as well as providing task-specific inputs to the *Generate ASO* module. For instance, splice-switching ASOs can be used to induce non-productive mRNA by skipping a particular exon, leading to a frameshift and the appearance of a premature termination codon. An additional module that, given a gene identifier, suggests which exon could be skipped to induce non-productive mRNA would be particularly useful, especially when feeding the results into the *Generate ASO* module. Similarly, ASOG would benefit from the implementation of a module for designing gapmers, which are particularly useful for RNase H-mediated ASOs. However, gapmers are based on a central DNA segment flanked by nucleotides with modified chemistry; hence, they are heterogeneous by nature and would require both specific input forms and extensive thermodynamic data to predict their hybridization temperature to the target. Finally, taking advantage of recent developments in machine learning approaches could help extend off-target and toxicity predictions beyond sequence similarity, which remains a challenging task due to the limited number of appropriate publicly available datasets for modeling.

Overall, we believe that ASOG makes ASO design more accessible to the community, while remaining free to use and user-friendly. In addition to facilitating the rapid exploration of ASO space, it encourages the standardization of ASO design workflows and provides community tools beyond the ASO generation module, thereby contributing to RNA biology research and therapeutic discovery pipelines.

## 6 Conclusion

We developed ASOG: Antisense Oligonucleotide Generator, a web server that automates ASO design and property assessment. It also provides standalone tools of broad interest for predicting melting temperatures and splice sites. ASOG facilitates efficient oligonucleotide design, enabling rapid exploration of the ASO space. Compared to existing tools for ASO design [9], [10], [11], [12], [13], ASOG adopts a generalist approach with explicit criteria for systematic ASO definition from a gene sequence. It calculates properties such as target topology, splice site locations, hybridization thermodynamics, self-folding, and off-target sequence detection, among others. ASOG supports fast searches and informed decision-making and is available at asog.iecb.u-bordeaux.fr.

## CRediT authorship contribution statement

**Jonah Kimi:** Writing – original draft, Software, Investigation, Formal analysis. **Patricia Korczak:** Validation, Methodology. **Brune Vialet:** Validation. **Eric Roubin:** Software. **Philippe Barthélémy:** Methodology, Funding acquisition, Conceptualization. **Sébastien Campagne:** Writing – review & editing, Resources, Project administration, Funding acquisition, Conceptualization. **Florian Malard:** Writing – original draft, Supervision, Software, Investigation, Funding acquisition, Formal analysis, Conceptualization.

## Declaration of competing interest

The authors declare that they have no known competing financial interests or personal relationships that could have appeared to influence the work reported in this paper.

## Data Availability

The ASOG web application is available at asog.iecb.u-bordeaux.fr. The source code was shared during the peer-review process for evaluation by reviewers.
